# A 3-year retrospective study of 866 children and adolescent outpatients followed in the Nice Pediatric Psychotrauma Center created after the 2016 mass terror attack

**DOI:** 10.3389/fpsyt.2022.1010957

**Published:** 2022-12-08

**Authors:** Morgane Gindt, Arnaud Fernandez, Radia Zeghari, Marie-Line Ménard, Ophelie Nachon, Aurélien Richez, Philippe Auby, Michele Battista, Florence Askenazy

**Affiliations:** ^1^Service Universitaire de Psychiatrie de l’Enfant et de l’Adolescent, Hôpitaux Pédiatriques de Nice Centre Hospitalier Universitaire-Lenval, Nice, France; ^2^Université Côte d’Azur, CoBTek, Fédération de Recherche Interventions en Santé, Nice, France; ^3^Centre Expert du Psychotrauma Provence Alpes Côte d’Azur Corse, Nice, France

**Keywords:** child and adolescent psychiatry, psychotrauma, taking care, PTSD, mental health

## Abstract

**Background:**

The mass terrorist attack in Nice, France, in July 2016 caused deaths and injuries in a local population, including children and adolescents. The Nice Pediatric Psychotrauma Center (NPPC) was opened to provide mental health care to the pediatric population (0–18 years) who experienced traumatic events.

**Objectives:**

This study describes the specificity of the care pathway for young trauma victims, with an explanation of how the NPPC works during the first three years.

**Methods:**

In this retrospective study, we conducted quantitative and qualitative data collection about new and follow-up consultations, primary and comorbid diagnoses, and the kind of trauma (terrorist attack versus other kinds of trauma). Ethics approval was obtained from the local Ethics committee.

**Results:**

866 children and adolescents were followed in the NPPC. We found a high rate of Post-Traumatic Stress Disorder (PTSD; 71%) in this population with a high rate of comorbidities (67%), mainly sleep disorders (34.7%) and mood and anxiety disorders (16.2%). A high number of children and adolescents impacted by the terrorist attack required follow-up consultations after exposure to the mass terrorist attack, the first care-seeking requests continued to occur three years later, although at a slower rate than in the first and second years. New consultations for other kinds of trauma were observed over time.

**Discussion:**

This study supports previous findings on the significant impact of mass trauma in the pediatric population showing even a higher level of PTSD and a high rate of comorbidities. This may be explained by the brutality of the traumatic event, particularly for this age group. The findings of this study have implications for early interventions and long-term care for children and adolescents to prevent the development of chronic PTSD into adulthood.

## Introduction

The mass terrorist attack in Nice (France), on the evening of the Bastille Day, on July the 14th, 2016, is one of several terrorist attacks that occurred in public places across European countries in recent years (1–4). This attack was perpetrated on French National Day, Bastille Day, traditionally celebrated with fireworks on the city’s renowned Promenade des Anglais. At the time of this incredibly violent event, more than 30,000 people were in the area, including many babies, children and adolescents (5). Eighty-six people died in the attack, including ten children, the youngest being four years old (6). At least fifty-five children and adolescents were bereaved because of this crime. This type of attack constitutes one of the highest existing traumatic stressors, with malevolent intent, extreme violence and harmfulness against unprepared victims. Terrorist attacks generate collective stress and maximal media coverage in the following period (7). In September 2022, several people accused of helping the assaulter killed during the attack went on trial in a special French terrorism court.

The most common diagnosis reported after traumatic events is Post-Traumatic Stress Disorder (PTSD) (8). After a terrorist attack, the rate of PTSD in the pediatric population is very high (between 50 and 75% of children) (9, 10). Re-experiencing symptoms, behavioral or emotional avoidance symptoms, cognition and mood alteration and arousal symptoms are the four main classes of PTSD symptoms (11). Some of these symptoms could affect the child’s learning capacities and quality of life (12–14). Moreover, children with PTSD could present an intense feeling of shame about the event, display regressive behavior (i.e., enuresis, encopresis, attachment problems with the caregiver and crying) or even report several psychosomatic complaints (15, 16). These different symptoms could alter their social and affective development. PTSD in children and adolescents could also lead to an alteration of personality, a high level of anxiety and a significant drop in self-esteem (17, 18).

PTSD in young children is often seen with comorbid disorders, almost in 75% of them (19). The most frequent are: Oppositional Defiant Disorder (ODD), Separation Anxiety and Attention Deficit Hyperactivity Disorder (ADHD). It has been reported that developing comorbid disorders influences the severity of PTSD symptoms and their sustainability (20–23). Nevertheless, a comparison of estimates of the burden of PTSD with the estimated cost of treating all adults with PTSD with the recommended treatments shows the potential for substantial economic gains to be made through extension and investment in effective evidence-based treatments (24). After a mass terrorist attack, children may also develop traumatic grief (25), mood disorders (26), anxiety disorders (27), behavioral disorders (28, 29), dissociative disorders (30) or psychotic episodes (31).

Consequences of psychiatric symptoms after a terrorist attack include a generalized decline in school performance (32), increased school absenteeism (33), and altered beliefs in the institutional system (justice or police) (34). In addition, children exposed to a terrorist attack have a higher risk of re-exposure to traumatic events (35). Exposure to traumatic experiences in childhood correlates with consequences in adulthood: psychiatric consequences (increased depression, anxiety disorders, and PTSD) (36, 37). After this event, a Pediatric Psychotrauma Center was created in January 2017 in Nice (Nice Pediatric Psychotrauma Center - NPPC) to offer psychotrauma assessment and specific care for children and adolescents exhibiting psychological symptoms (38). NPPC offers personalized and multidisciplinary pediatric care. The staff comprises a large panel of professionals, physicians (child and adolescent psychiatrists and pediatricians), psychologists, occupational therapists, nurses and social workers. NPPC is one of the two leaders of the PACACorsica Psychotrauma Center, and part of the 15 centers in France labeled “Psychotrauma” (39). Ever since, our missions are multiple: emergency psychotraumatic care; medium and long-term psychotraumatic care; training students in psychology, medicine, sophrology; University teaching (Psychology department, Medicine Department, university degree in Psychotrauma); vocational training for child and adolescent psychiatrists, psychologists, social workers, educational professionals, justice departments, emergency unit volunteers etc.; research activities involving several international scientific collaborations and scientific communications. This study aims to highlight the specificity of the care pathways for child and adolescent trauma victims, with an explanation of the functioning of the NPPC during the first three years, as well as a description of the patients who were received and managed within the NPPC.

## Materials and methods

From January 2017 to December 2019, qualitative and quantitative data were retrospectively collected from the NPPC patient’s active file and medical records (at the Children’s Hospitals of Nice CHU-Lenval): (1) Potential traumatic events (DSM 5), (2) DSM-5 diagnoses (main diagnosis and associated diagnosis), (3) New consultations, and (4) Number of follow-up consultations.

The list of traumatic events experienced by the child, as well as the diagnoses (principal and associated) are recorded at the end of the new consultations in the patient’s computerized medical record. New consultations are primary consultations in which a child psychiatrist and/or a psychologist evaluates the patient to decide the treatment pathway and refer to a psychological follow-up for instance. For each patient there is only one further consultation, even if the child relives a traumatic event. Follow-up consultations are consultations with a psychologist, psychomotor therapist or psychiatrist where patients are seen regularly (e.g., every week or two weeks, every month…). The number of follow-up consultations are the number of time a patient have been at the centre and saw their therapist.

The study has been carried out per the 1964 Declaration of Helsinki and its later amendments and declared to the French National Commission on Informatics and Liberty (CNIL). Ethics approval was obtained from the local Ethics committee (n° 2021-064).

### Participants

During the three years since the creation of NPPC, 752 patients came for a new consultation and 866 patients consulted for a follow-up in the NPPC (Table 1). The difference between these two figures is related to the fact that some children were already in regular follow-up when the NPPC was created and continued their care without having a new consultation. The mean age of this pediatric population is 10 years and 4 months (standard deviation = 4.7; ranging from 6 months of age to 19 years). The distribution of the age variable follows the normal distribution (chi-square test = 122, *df* = 8, *p* < 0.001). Caseload is made up of 51% girls and 49% boys.

**TABLE 1:** NPPC consultations per year.

	2017		2018		2019	
			
	New	Follow-up	New	Follow-up	New	Follow-up
Terrorist attack	226	251	175	308	68	312
Other kind of trauma	55	75	82	129	146	217
Total	281	326	257	437	214	529

### Statistics

Descriptive analyses (percentage, mean and standard deviation) were performed on the number of new and follow-up consultations as well as for the primary diagnoses, comorbidities and the nature of the trauma.

Repeated measures ANOVA were used to perform analyses. We compared the number of new consultation for the terrorist attack and the other kind of trauma per year. We analyzed the evolution over the years of the number of follow-up consultations for the terrorist attack and other kinds of trauma. The effect sizes were expressed as partial eta-squared values within repeated measures ANOVA squared (pes; small ≥ 0.01, medium ≥ 0.06, large ≥ 0.14).

## Results

### Reasons for consultation

**FIGURE 1:**
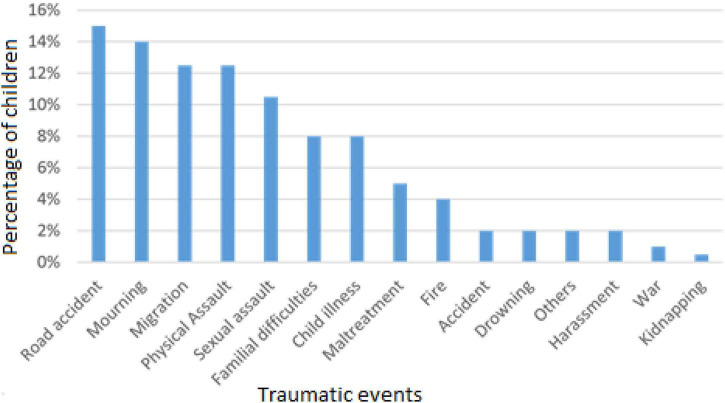
Other kinds of trauma from 2017 to 2019 (*N* = 337).

Among 866 patients, 529 consulted for the terrorist attack (61.1%) and 337 for other kinds of trauma (38.9%; Figure 1), the most frequent being road accidents (15%), mourning (14%), migration (12.5%), physical and sexual assault (12.5% and 10.5%).

The majority of these patients were diagnosed with PTSD (71%) or with acute stress disorder (9%) by clinicians. The complete list of main diagnoses is presented in Table 2.

**TABLE 2:** Main diagnoses (DSM-5) from 2017 to 2019 (*N* = 866).

Main diagnoses	
PTSD	71% (615)
Acute stress disorder	9% (78)
Others	8.8% (76)
Anxiety disorder	7.3% (63)
Neurodevelopmental disorders	2.4% (21)
Major depressive disorder	1.5% (13)

% (*n*).

Children and adolescents with PTSD have a high comorbid rate (67%). Table 3 summarizes the main comorbidities. Sleep-wake disorders, anxiety and neurodevelopmental disorders are the three most common comorbidities, accounting for 75%.

**TABLE 3:** Main comorbidities (DSM-5) associated to PTSD (*N* = 412).

Main PTSD comorbidities	
Sleep-wake disorders	34.7% (143)
Anxiety disorders	27.6% (114)
Neurodevelopmental disorders	12.9% (53)
Somatic symptom and related disorders	8.0% (33)
Disruptive, impulse control and conduct disorders	6.1% (25)
Others	3.8% (16)
Elimination disorders	3.0% (13)
Dissociative disorders	2.5% (10)
Feeding and eating disorder	1.2% (5)

% (*n*).

### New consultation

**FIGURE 2:**
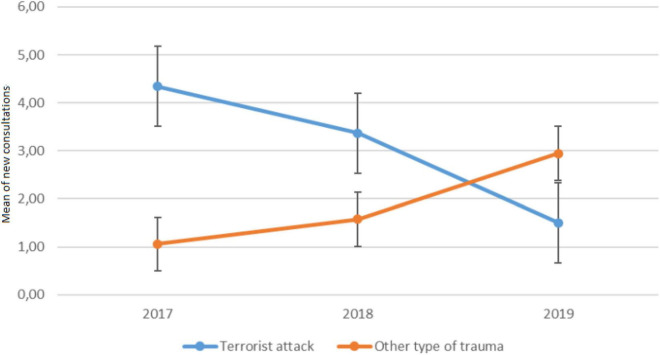
Mean numbers of new consultations per week according to the type of trauma and time.

Regarding new consultation (see Figure 2), the repeated measures analysis of variance, performed between group (attack versus other kind of trauma) and time (2017 vs. 2018 vs. 2019) was significant [*F*(2, 204) = 27, *p* < 0.001]. New consultations differed by year and type of trauma (*p* < 0.001, *pes* > 0.49, large). In 2017, the number of new visits was significantly greater for the terrorist attack group (mean = 4.3, SD = 2.7) than for the other kind of trauma group [mean = 1.1, SD = 1.3; *F*(1, 102) = 62.8, *p* < 0.001]. The same results were obtained for the year 2018, with more new patients for the terrorist attack group (mean = 3.4, SD = 3.3) than for the other kind of trauma group [mean = 1.6, SD = 1.7; *F*(1, 102) = 11.7, *p* < 0.001]. In 2019, the number of new consultants was greater for the other kind of trauma group (mean = 2.9, SD = 2.5) than for the terrorist attack group [mean = 1.5, SD = 1.8; *F*(1, 102) = 11.4, *p* < 0.001].

### Follow-up consultations

**FIGURE 3:**
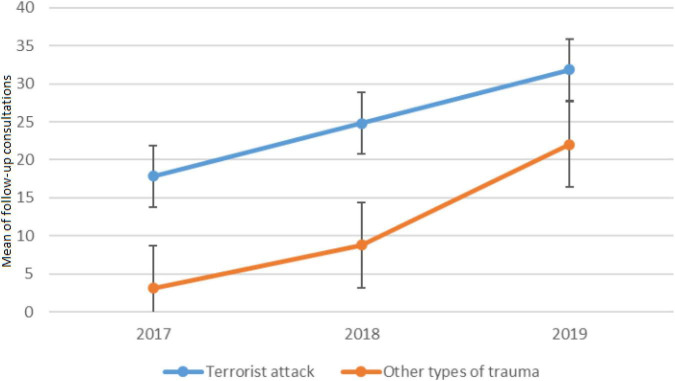
Mean number of follow-up consultations per week according to the traumatic event and time.

Regarding the interaction between group and time for follow-up consultations, the result was significant [*F*(2, 204) = 4.15, *p* < 0.05, see Figure 3]. Follow up consultations increased during the three years according to the type of trauma (*p* < 0.001, *pes* > 0.64, large). In 2017, the majority of follow-ups involved patients who experienced the terrorist attack (mean = 18, SD = 8.6) compared with other kinds of trauma [mean = 3, SD = 2.7; *F*(1,52) = 138.6, *p* < 0.001]. Similar results were found for 2018 and 2019. The number of follow-ups was significantly greater for the terrorist attack (mean = 25 and 32, SD = 11.8 and 3.3) than for the other kind of trauma [mean = 8.8 and 22, SD 6.4 and 10.4; *F*(1,52) = 74, *p* <.01 and *F*(1,52) = 17.4, *p* < 0.001]. For the terrorist attack group, there was an increase in follow-up consultations from 2017 to 2018 [*F*(1, 52) = 23.4, *p* < 0.001] and from 2018 to 2019 [*F*(1, 52) = 58.8, *p* < 0.001]. The same results were obtained for other kinds of trauma with an increase between 2017 and 2018 [*F*(1,52) = 15.5, *p* < 001] for follow-up consultations and between 2018 and 2019 [*F*(1, 52) = 75.6, *p* < 0.001].

## Discussion

This paper aims to present data related to the activity of the Nice Pediatric Psychotrauma Center (NPPC) three years after the unit’s inception.

866 children and adolescents were treated at the NPPC. For the great majority, the traumatic event, which led to seeking consultation, was related to the terrorist attack on the 14th of July in 2016. Patients with other types of traumatic events (road accidents, mourning, migration, etc.) were also treated at the NPPC. The primary diagnoses recorded were PTSD (71%), but other diagnoses such as acute stress disorders, major depression disorder, and anxiety disorder were also recorded. In fact, patients with PTSD had a high comorbid rate such as sleep-wake disorders, anxiety and neurodevelopmental disorders.

Potentially traumatic experiences are common in children and adolescents: 65% of children aged 6–12 experience a trauma; this percentage reaches 81.5% for those aged 16–18 (40), and that more than a third had experienced multiple traumas (41). Among the children followed in NPPC, road accidents, mourning, traumatic migration, physical and sexual assault are the most frequent traumatic events, apart from the terrorist attack of July 14, 2016. Exposure to a traumatic event as a child is consistently associated with more severe health and mental health problems than adult exposure (42, 43). The risk of chronicization of this pathology is around 25% in children (44–47), and relapse rate is as large as 40% (48, 49).

During the first three years of activity of NPPC, we observed 9% of acute stress reactions and 71% of PTSD, in line with the literature, reporting a prevalence of PTSD between 20 and 50% after traumatic exposure (50–54). Several factors are implicated in the development of PTSD. Interpersonal and repeated events cause three times more PTSD than non-interpersonal and single events (55). Age is also known to have an influence on the development of trauma disorder: the risk of PTSD after a potentially traumatic event is about 39% for preschool children and 27% for adolescents (19).

Among the comorbidities associated with PTSD in our population, sleep disturbances are common, whether they are nightmares or difficulty falling asleep. These results are consistent with studies carried out on the association between psychotrauma and sleep disorders in children (56–59). Moreover, symptoms of PTSD may be internalizing and externalizing in trauma-impacted children (60, 61). Externalized behavioral symptoms may lead to oppositional defiant disorder, conduct disorder and aggression, while internalized behavioral symptoms refer to somatic complaints, depression or anxiety (62). In children, comorbid PTSD disorders are estimated at 75% (63). It can be depression, separation anxiety, generalized anxiety with panic attacks, attention deficit disorder or mood disorders (19, 64, 65). Studies evaluating the long-term impact of traumatic events in children and adolescents, such as natural disasters, indicate a significant rate of PTSD associated with other anxiety disorders or mood disorders (66–68). A comorbid disorder appears to influence PTSD symptomatology intensity and maintenance (22–24).

The evolution of the number of new and follow-up consultations was also recorded. From 2017 to 2018, most new consultations and follow-ups at the NPPC were related to the terrorist attack of 2016. However, in 2019, new consultations were related to other types of traumatic events. Both numbers of new consultations and follow-ups increased significantly over the years. This can be explained by several factors, including the notoriety of the center, both for professionals and the public, as well as the integration of the NPPC into the care pathways offered by the children’s hospital. Indeed, the creation of this center has allowed children and families who are victims of a road accident, requiring a visit to the hospital (emergency department, intensive care unit or somatic department) to have access to a team trained in psychotrauma from the very beginning.

The results of our study highlight that the follow-up of patients seen for the terrorist attack may extend over several years and require more follow-up consultations than patients seen for other types of trauma. This result can be explained by several hypotheses including the terrorist act itself. Indeed, intentional and violent trauma are known to be the most PTSD-inducing experiences, which for 39% of individuals with PTSD, show a chronic course of the condition (69, 70). Another explanation is related to the implementation of care pathways and access to care for populations affected by the attack. Currently, new consultations are still being carried out for the terrorist attack. No psychological care has been undertaken before. The pathologies are therefore chronic and strongly associated with each other, which requires a more global and longer care. In most European countries, scientific societies related to stress and psychotrauma have been created to promote good clinical practices and develop training in psychotrauma (71). Guidelines for the management of trauma exist: for example, World Health Organization (72) and American Academy of Child and Adolescent Psychiatry (73) recommend early interventions for children and adolescents to reduce PTSD clinical manifestation. Early-onset chronic stress does not heal naturally, and its effects appear to exacerbate over time (74). Therefore, intervening early is crucial to minimize and reduce long-term negative effects (75). Early intervention aims to avoid PTSD chronicity. The chances of responding favorably to therapy decrease once the PTSD become chronic (76, 77). After such mass trauma, our experience at the NPPC underlines the need for a clinical team that can handle a massive influx of children and provide long-term follow-up. As suggested by the PTSD Guideline Development Group and the National Collaborating Centre for Mental Health, social and psychological care must range from immediate comfort and practical help to longer-term psychological support, which may last 18–24 months or more (78).

In addition, after a mass trauma, the NPPC experience highlights the need for specialized pediatric consultations and targeted communication to ensure population awareness of these resources (5). Moreover, the first consultations still occur, which was unexpected and is an exciting finding to report, even if their number decreases over time, up to three years after the event. Several hypotheses may explain this phenomenon, including the appearance of delayed symptoms or avoidance behaviors. The difficulty in accessing care must be taken into account as soon as possible after the traumatic event in order to avoid therapeutic wandering and to promote a proactive organization of care. PTSD and comorbidities can persist for decades (8). According to these authors, trauma-focused treatment and monitoring for chronic medical illnesses are essential components of recovery programs. Following AACAP recommendation, Psychologists of NPPC provide Cognitive-Behavioral Therapy (CBT) and Eye Movement Desensitization and Reprocessing (EMDR), which appears to reduce significantly the fear and general worryness in adolescents. CBT exhibits the most significant results (79, 80). CBT can be performed with both children and adults, and has proven to be efficacious for a large spectrum of psychiatric disorders, especially for the treatment of anxiety disorders (81, 82).

The analysis of the NPPC activity also highlights the need to take care of any trauma in the pediatric population. Due to the improved visibility of the NPPC after three years, requests for initial consultations have increased each year steadily. To respond to the increasing number of new and follow-up consultations, new health care and social professionals, including those with additional background, joined the NPPC team.

### Study limitations

One limitation of this study is that the specificities of the care pathways implemented at NPPC according to developmental age and the individual clinical features of each child are not described and statistically analyzed.

Another limitation is the lack of comparison of key diagnoses and comorbidities by kind of traumatic event: terrorist attack versus other traumatic events versus multiple events. It was impossible to make such a comparison on the first three years’ data; however, this comparison will be performed for the longer term as the study is ongoing.

## Conclusion

The unique experience of the NPPC highlights the need for a specific emergency psychological response and the need for specialized multidisciplinary follow-up consultations for children, adolescents and their families after a traumatic event. The type of traumatic experience must be taken into account in the care of the child and his family. The more violent and intentional the experiences, the more intense and heterogeneous the symptoms may be and the longer the care required. Standardized and regular assessments seem necessary to provide personalized care of children according to their age, development and symptoms.

The NPPC experience highlights the need for health policies to create centers dedicated to the care of children and families impacted by a traumatic event to avoid long-term consequences, including in adulthood.

Further studies are needed to better evaluate the impact of specific versus non-specific care, depending on the traumatic events experienced and the developmental ages of the young people.

## Data availability statement

The raw data supporting the conclusions of this article will be made available by the authors, without undue reservation.

## Ethics statement

The studies involving human participants were reviewed and approved by CERNI No: 2021-064. Written informed consent to participate in this study was provided by the participants’ legal guardian/next of kin.

## Author contributions

MG and AR realized the statistics. All authors participated in the writing and proofreading of this article.
